# Lineage grammars: describing, simulating and analyzing population dynamics

**DOI:** 10.1186/1471-2105-15-249

**Published:** 2014-07-21

**Authors:** Adam Spiro, Luca Cardelli, Ehud Shapiro

**Affiliations:** Department of Computer Science and Applied Mathematics, Weizmann Institute of Science, Rehovot, Israel; Microsoft Research, Cambridge, UK; Department of Biological Chemistry, Weizmann Institute of Science, Rehovot, Israel

## Abstract

**Background:**

Precise description of the dynamics of biological processes would enable the mathematical analysis and computational simulation of complex biological phenomena. Languages such as Chemical Reaction Networks and Process Algebras cater for the detailed description of interactions among individuals and for the simulation and analysis of ensuing behaviors of populations. However, often knowledge of such interactions is lacking or not available. Yet complete oblivion to the environment would make the description of any biological process vacuous. Here we present a language for describing population dynamics that abstracts away detailed interaction among individuals, yet captures in broad terms the effect of the changing environment, based on environment-dependent Stochastic Tree Grammars (eSTG). It is comprised of a set of stochastic tree grammar transition rules, which are context-free and as such abstract away specific interactions among individuals. Transition rule probabilities and rates, however, can depend on global parameters such as population size, generation count, and elapsed time.

**Results:**

We show that eSTGs conveniently describe population dynamics at multiple levels including cellular dynamics, tissue development and niches of organisms. Notably, we show the utilization of eSTG for cases in which the dynamics is regulated by environmental factors, which affect the fate and rate of decisions of the different species. eSTGs are lineage grammars, in the sense that execution of an eSTG program generates the corresponding lineage trees, which can be used to analyze the evolutionary and developmental history of the biological system under investigation. These lineage trees contain a representation of the entire events history of the system, including the dynamics that led to the existing as well as to the extinct individuals.

**Conclusions:**

We conclude that our suggested formalism can be used to easily specify, simulate and analyze complex biological systems, and supports modular description of local biological dynamics that can be later used as “black boxes” in a larger scope, thus enabling a gradual and hierarchical definition and simulation of complex biological systems. The simple, yet robust formalism enables to target a broad class of stochastic dynamic behaviors, especially those that can be modeled using global environmental feedback regulation rather than direct interaction between individuals.

## Background

In recent years there has been a great interest in modeling and simulating various aspects of population dynamics in biological and ecological systems [1–4]. The increasing computational resources along with a deeper understanding of biological and ecological phenomena have led to the development of many languages for describing, analyzing and simulating concurrent stochastic processes. Many such languages specify Markovian dynamics and differ by level of abstraction, ease and complexity of the description and execution efficiency [5]. Two widely used formalisms are based on Chemical Reaction Networks (CRN) [6] and stochastic Process Algebras (PA) [7].

CRNs were originally used to describe chemical systems. A CRN description consists of a finite set of reactions acting on a finite number of species. Each reaction specifies the identity and stoichiometry of the reactants and products along with a rate constant. Many processes can be described using CRNs, for example, Predator-Prey models [8], Cellular cascade pathways [9], Cancer progression [10], Epidemics dynamics [11], and many others [1]. Each of these processes consists of a continuous interaction between individual species (the reactants) that occurs at a certain rate and produces a group of other individuals (the products, which may be empty) that can be of the same (autocatalytic) or of different type. The description of dynamical systems using CRN is relatively simple and can be used both for analytical solving and simulations. However, this approach neglects biological aspects of the described systems by treating each object (reactant or product) as a simple entity, which ignores its environmental context and structure. For example, many molecular objects maintain their overall identity while changing in specific attributes, such as chemical modification or location. When using a CRN abstraction such molecules cannot retain identity while changing state.

PAs, on the other hand, are a family of mathematical formalisms that were originally developed to model concurrent computer systems. They enable the abstraction and specification of communication and synchronization between a collection of processes by passing messages between them. One of the most well studied PA is the *π* -calculus, which has been shown to be very useful in describing a range of biological systems [7, 12]. The language consists of processes that are mapped to real-world objects, and channels, which are mapped to communications and interactions between the different objects. A unique feature of the *π* -calculus allows to dynamically communicate new channels between the processes (this is termed *mobility*), which enables the objects to keep their identity while changing their internal states or interactions with other objects. This feature is more compatible with real biological and ecological scenarios and fits well to the way we think and observe these processes. It also allows one to abstract and specify the dynamics in a more accurate fashion. It has also been shown that this abstraction can be treated as an executable computer program, allowing to stochastically simulate any specified model [13].

Many tools have been developed in order to allow and simplify the use of mathematical modeling for the life-science community, and each one has its strengths and weaknesses [14–16]. There is no single formalism that has all the required features and choosing the appropriate one depends on the specific goals and resources of the modeler. Our goal in this work is to develop and formulate a simpler and practical tool for modeling and simulating the behavior and interaction of populations. We do so by extending the notion of Stochastic Tree Grammar (STG) [17] by incorporating both rates and probabilities to the transition rules. These can be dynamically updated by defining them as functions of the system’s state, which includes global values such as current population size, generation count or elapsed time. In addition, we extend the system by allowing each individual to hold its own internal states which can change through inheritance. We later discuss implementation of stochastic simulation and the relation to Ordinary Differential Equations (ODE).

A prominent feature of the language is that it enables to stochastically produce possible lineage trees corresponding to single executions. These lineage trees contain a representation of the entire events history of the process, including the dynamics that led to the existing as well as to the extinct individuals. As opposed to standard approaches that output only the population size dynamics, our implementation also outputs the corresponding lineage trees, which can be used to analyze the evolutionary and developmental history of the process.

Recently, Vaughan et al. [16] presented the usage of CRNs as lineage grammars and used them to simulate phylogenetic trees. Although they enable to sample possible genealogies based on the defined reaction rules, they do not allow the specification and analysis of more complex behaviors such as feedback onto the dynamic rates and general inherited properties.

Throughout the paper, we demonstrate the usability of the language by presenting a wide range of examples that can be modeled and simulated using this approach. The examples show that the language can provide simple descriptions of systems from various domains. Example parameter values were taken from the literature when available or chosen arbitrarily in order to simplify the presentation.

## Results and discussion

### eSTG programs

Following is an example of an eSTG program for stem-cell differentiation [18]:


In this example, *SC* (stem cells) divide symmetrically 0.1 times per day, while self-renewing or differentiating with the same probability (50%), and *Diff* (differentiated cells) can once a day either proliferate (with probability 49%) or die (with probability 51%).

**Figure 1:**
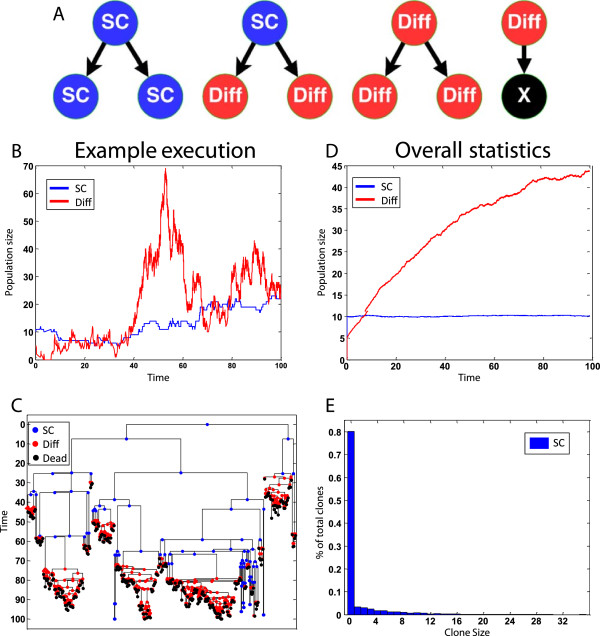
**An example of the stem cell differentiation program execution.** The program was executed up to simulation time 100 days. **(A)** Schematic representation of the eSTG rules (without rates and probabilities). **(B)** Population size over time of a specific execution. **(C)** Cell lineage tree of a specific execution (only one cell lineage tree out of the originating *SC*s and *Diffs* is shown). **(D)** Average population size over time (calculated from 1000 stochastic executions). **(E)** Clone size distribution, which is the final population size derived from each initiating individual (calculated from 1000 stochastic executions).

Alternatively, one can define an average time to event *t* instead of a rate, which can be translated interchangeably into a rate using . The above rules are then written:


An execution of an eSTG program proceeds through the stochastic application of its transition rules on its state. An example execution of the program, on an initial 10 *SC* and 5 *Diff*, can be summarized by a cell lineage tree and population size graphs shown in Figure 1B and Figure 1C. In addition to single executions, eSTG can also be used for obtaining overall population statistics, for example, to calculate the average population size over time (Figure 1D) and the distribution of clone sizes (Figure 1E).

**Figure 2:**
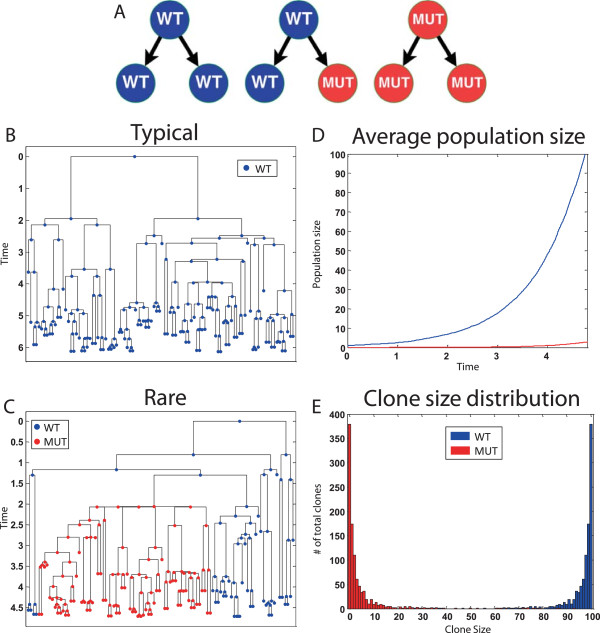
**An example of the Luria–Delbrück program execution.** The program was executed from 1 ***WT*** to 100 cells. **(A)** Schematic representation of the eSTG rules. **(B)** Typical lineage tree execution where mutations do not occur early. **(C)** Rare lineage tree execution where a mutation occurs early. **(D)** Average population size over time (calculated from 1000 stochastic executions). **(E)** Clone size distribution (calculated from 1000 stochastic executions). In the rare events where the mutation happens early in the lineage, the clone size of the mutated population is large.

Following is another example of an eSTG program for the Luria–Delbrück Model [19]:


In this model, wild-type bacteria (*WT*) are randomly mutated (in the absence of selection) to form a resistant bacteria (*MUT*), thus the population size of mutated bacteria varies dramatically and is dependent on the timing in which the mutation has happened. Figure 2B and Figure 2C show specific executions of typical and rare lineage trees. Averaging over many executions can yield average population size (Figure 2D) and clone size distribution (Figure 2E).

### Internal states

We define internal states for each species as a vector of variables that can change, either deterministically or stochastically for each individual, with every execution of a rule. Internal states can be used to model inherited attributes, such as mutations or substance accumulation, or record historical events such as the number of generations, number of symmetrical/asymmetrical divisions, or time since historical events. We thus extend the basic rules defined above to include internal states which are functions of the predecessor’s internal states. For example, extending the previous stem-cell differentiation scenario:


In this example, we define a vector of *n* variables , which correspond to the number of repeats in *n* Microsatellite (MS) loci in the DNA [20]. In every cell division, the number of MS repeats for each locus changes according to the stochastic function *f*_*MS*_, which can cause either a decrease or an increase of one repeat with probability *p*[21]:


This simulated data can be used for example to evaluate the relationship between *n*, the number of MS, and the accuracy of phylogenetic reconstruction based on MS lengths of the tree (see [22, 23] for details).

**Figure 3:**
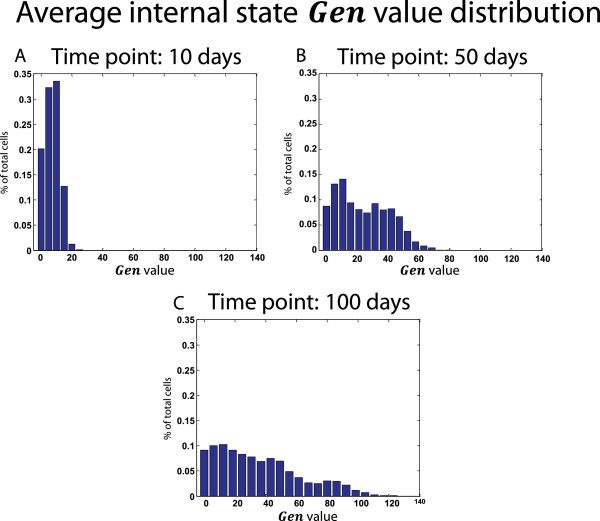
**An example of generation counter internal state.** Each species of the type *Diff* holds an internal state called ***Gen*** which holds the number of cell divisions since the differentiation event. The histogram of the ***Gen*** values over the entire population can be calculated at different time points (e.g. after 10, 50 and 100 days, shown in **(A)**, **(B)** and **(C)** respectively).

Another example for the use of internal states is the following program, which counts the number of generations since each differentiation event:


Figure 3 shows various distribution statistics of the internal state *Gen* over the population at different time points.

Other examples of internal states can be the counting of historical events (such as how many symmetric vs. asymmetric divisions a cell went through) or measuring the time since a certain event.

### Probabilities and rates as functions

Population dynamics can change based on various conditions such as population size, internal or external changes, and elapsed time. A common phenomenon in population dynamics is the reaching of a homeostasis, meaning that at a certain point, the population size reaches a steady state.

A simple example is the growth of a species until reaching a target size. Consider the following parametric rule:


Without feedback regulation on the population size, a setting of  results in an extinction with probability 1 [24]. A simple regulation scheme is the logistic model [25]:


where *N* is the population size, *r* is the growth rate and *K* is the target size (also termed carrying capacity). We can use the above parametric eSTG rule to model a logistic population growth by solving:

 (we use *A* as the population size of the species *A*)


For simplicity, *r* in the eSTG rule is the same as the *r* in the logistic model.

**Figure 4:**
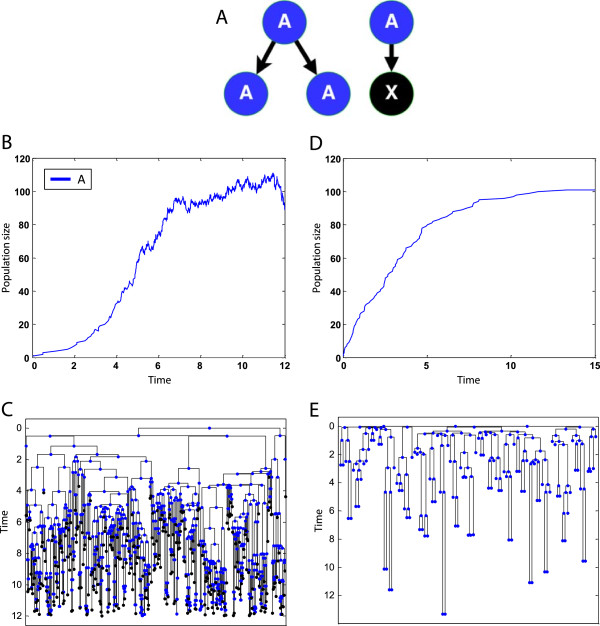
**An example of dynamic population growth.** An example of a simple proliferation with fate probabilities and rates that are functions of the population size. **(A)** Schematic representation of the eSTG rules. **(B)** Population size over time of a logistic growth starting from a single instance. **(C)** The corresponding lineage representation of the specific execution. **(D)** Population size over time of a production-removal growth starting from a single instance. **(E)** The corresponding lineage representation of the specific execution.



We then get:


Figure 4B and Figure 4C show the resulting dynamics (population size and lineage tree) starting from a single *A* of the following program (setting *K* = 100):

In a different scenario, the growth is also regulated by the rate but is leading to the same steady state. Using the following production-removal equation [26]:


we can model the dynamics using the parametric eSTG by solving:


The steady state of this system is  and for simplicity we limit *p* to be either 0 or 1, and set *α* = 1, *β* = 100. We thus define the following eSTG program:


Here, the rate is inversely dependent on the population size and the population is growing until reaching the steady state that is maintained by a feedback on *p*, which causes either a proliferation (*p* = 1) or death (*p* = 0). Figure 4D and Figure 4E show the resulting dynamics starting from a single *A*.

**Figure 5:**
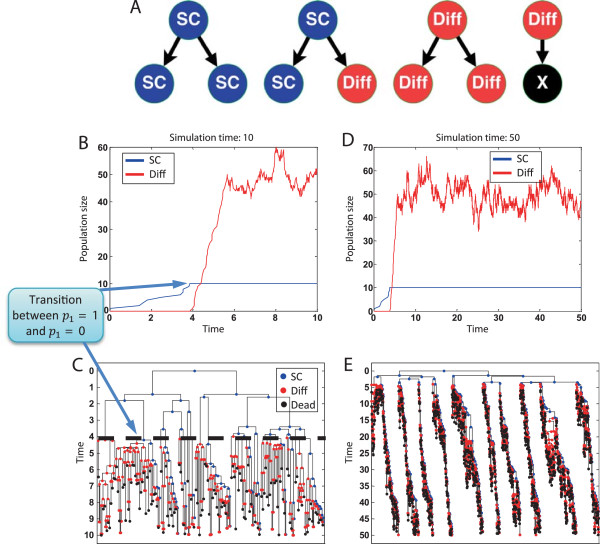
**Rules for optimal development of the crypt.** Simulation results of the rules for optimal development of the crypt (see main text). The rules are executed with *r*_1_ = 1.07, *r*_2_ = 1, |*SC*|_*Time* = 0_ = 1, |*Diff*|_*Time* = 0_ = 0, |*SC*|_*Target*_ = 10, |*Diff*|_*Target*_ = 50 (values are taken from [27]). Shown are execution results for two time windows starting with one *SC*. **(A)** Schematic representation of the eSTG rules. **(B)** Population size for simulation time of 10 days. The beginning of the process is shown where the switch between *p*_1_ = 1 and *p*_1_ = 0 occurs at around time *t* = 3.6. **(C)** The corresponding lineage representation of the specific execution. **(D)** Population size for simulation time of 50 days. Shown is the homeostatic phase that occurs after |*Diff*|_*Time* = *t*_ reaches |*Diff*|_*Target*_ at around time *t* = 6. **(E)** The corresponding lineage representation of the specific execution. It is interesting to observe the 10 clones that are maintained by the 10 *SCs*.

Another interesting scenario is described in [27], where an optimal development of the intestinal crypts is analysed. In the first stage, stem-cells are quickly amplified using self-replicating symmetric divisions, and after reaching the target size they differentiate asymmetrically into stem-cells and differentiated cells. We can describe this scenario using the following rules:


where |*X*|_*Time* = *t*_ is the population size of species *X* at time *t* and |*X*|_*Target*_ is the target population size of *X*. Although not described in [27], we continue the scenario with homeostasis by solving:


We thus extend the program with the following:


Figure 5 shows simulation results of a specific execution.

An example from a different regime is the predator/prey model of Lotka-Volterra [8]. It describes the interaction dynamics between two species using two ODEs:


where *c*_*i*_ are parameters. These equations are usually translated into the following mass action kinetic reactions:


Since eSTG has only context-free transitions, we convert the second reaction into two unimolecular reactions while preserving the 2^nd^ order rate (see Methods for a general method to convert CRNs to unimolecular reactions while preserving the same underlined ODEs). The new reactions and their rates are described in Table 1. We note that although these new reactions are not identical to the original ones, they are still in agreement with the ODEs described above. The model can be described using the following parameterized eSTG program:

**Table 1:** **Lotka-Volterra unimolecular representation**

Reaction	Global rate
*Prey* → 2*Prey*	*c*_1_ ⋅ *Prey*
*Prey* → *ϕ*	*c*_2_ ⋅ *Prey* ⋅ *Predator*
*Predator* → *2Predator*	*c*_2_ ⋅ *Prey* ⋅ *Predator*
*Predator* → *ϕ*	*c*_3_ ⋅ *Predator*



**Figure 6:**
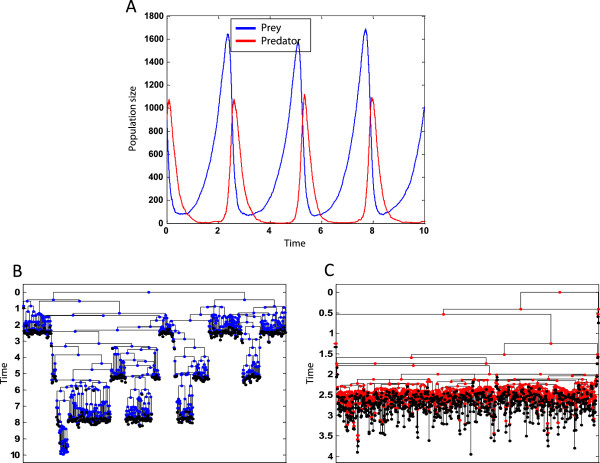
**An example execution of the Lotka-Volterra scheme.** An output example of the executed program using *c*_1_ = 2, *c*_2_ = 0.01, *c*_3_ = 5, |*Prey*|_*Time* = 0_ = |*Predator*|_*Time* = 0_ = 900. **(A)** Population size as a function of time. **(B)** A lineage tree of one of the 900 originating preys. **(C)** A lineage tree of one of the 900 originating predators. Both **(B)** and **(C)** exhibit the characteristic bottleneck phenomenon, where most lineages get extinct.

Figure 6 shows an example execution of the program.

The role of different feedback strategies on the control of organ and tissue growth can be investigated through the rates and probabilities of cellular decisions. Lander et al. [28] suggest two types of feedback strategies for the Olfactory Epithelium, one on the rate of division and the other on the probability of self-renewal (while keeping a constant division rate). They show that a feedback control onto the probability is a much more effective strategy for steady-state robustness and rapid regeneration.

The two strategies can be described using the following eSTG program (*SC* – Stem Cells, *INP* - Immediate Neuronal Precursor, *ORN* - Olfactory Receptor Neuron):


and the two feedback strategies are implemented by updating the *INP* parameters.

Strategy 1: Feedback onto the probability

 where *g* is a constant.

Strategy 2: Feedback onto the rate:

 where *h* is a constantFigure 7 shows possible executions generated using the two suggested strategies.

**Figure 7:**
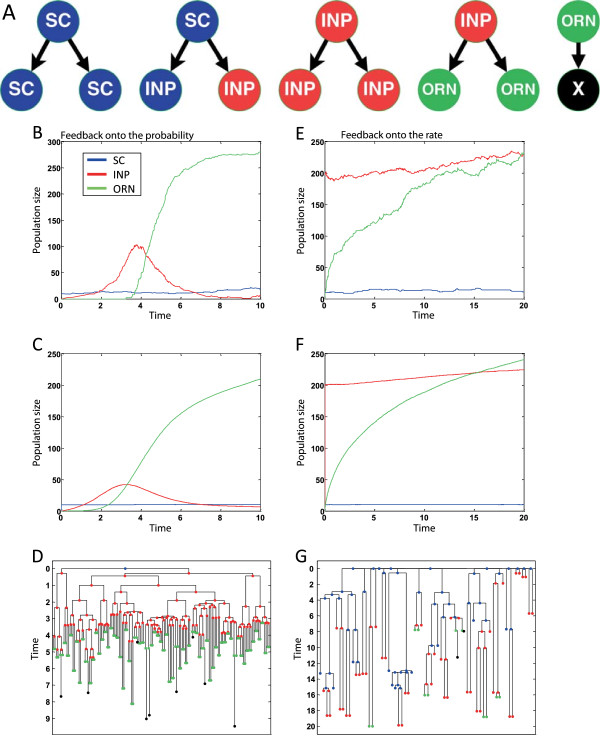
**Scenarios for feedback regulation. (A)** Schematic representation of the eSTG rules. Left plots - Feedback regulation onto the probability, where population size of an example execution, average population size over 1000 executions and an example of a lineage tree starting from a single *SC* are shown (**B**, **C**, and **D** respectively). Execution started with 10 *SC* *s*,  simulation time: 10. Right plots - Feedback regulation onto the rate, where population size of an example execution, average population size over 1000 executions and an example of a lineage tree starting from 10 *SC* *s* are shown (**E**, **F**, and **G** respectively). Execution started with 10 *SC* *s* and 200 , simulation time: 20 (values are taken from [28]).

### Possible extensions

#### Compartments

In many cases the population moves stochastically between different compartments, where each compartment corresponds to a different environment and different resources. Extending the language to include compartments allows one to define the same transition rules for species from the same type but different rates and probabilities, depending on the physical location of the individual. The system’s state is then extended to include the population size in each compartment. In addition to the regular transition rules, one also needs to define rules for the migration of each species between each two compartments.

#### Individual’s probabilities and rates as functions

Defining probabilities and rates for each individual separately is not recommended due to heavy computational requirements when implementing such a scenario, however, an extension of the language can support such a definition. In this case we can allow the probabilities and rates of each individual to be also dependent on its internal states. This allows each individual to have a distinct stochastic value of its probabilities and transition rates. For example, we can define a more sophisticated predator/prey model where the probability of reproduction is dependent on the individual’s age (or weight) which is represented as an internal state, or define the proliferation dynamics of a cell based on its mutations (represented as internal states).

## Conclusions

Stochastic simulation is a powerful tool to execute a complicated modeling system for which a closed form analytical solution is not possible. In addition, a simulation can generate a sample of representative scenarios that can be used for further analysis or as inputs to other programs. The complexity of natural phenomena requires a formal description framework which on one hand should be rich enough to capture the complexity and dynamics of the system and on the other hand will be compact and simple so it can be widely used by a broad community and could be implemented efficiently. There are many systems that are purely generative and derive their core results by ignoring interactions (e.g. L-Systems [29] and branching processes [24]). Although the assumption of independence enables certain analytical techniques, it precludes the ability to model processes and lineages that evolve through complex interactions between individuals and their environment. In order to allow both generativity and interaction, systems such as PA and CRN are more suitable. As described in [3], the trend towards individual-based stochastic models carries many advantages; they are easier to construct, more intuitive and can predict richer phenomena than population level models. In addition, it is possible to deduce population level conclusions (such as the underlined ODEs, see Methods) from the stochastic model. The presented formalism does not offer a new modeling approach in the sense that eSTG programs can be translated interchangeably into other languages (see Methods). Instead, the suggested eSTG language formalism allows a simpler description and specification of complex stochastic dynamics of individual entities. As demonstrated by the host of examples provided, these may include population level feedback from the current system’s state (either population size, internal or external factors) onto the rates and probabilities of the different species. In addition, eSTG, as a lineage grammar also enables the representation and analysis of historical events including those of extinct sub-lineages and transitional time points. Derivation trees produced by simulations can be examined for consistency with specific biological hypotheses [22, 30], so that eSTG models can be validated or falsified on the basis of the trees that they generate.

The language can also be used as a basis for inference and learning of the system’s governing rules, described in the eSTG formalism as the transition rules and the underlying rates and probabilities as functions of the system’s state. The question of parameter inference from biological data is an active area of study [31–34]. In our context, biological knowledge inferred from experimentally-obtained trees [22, 30, 35–43] could be used in order to infer the corresponding lineage grammars [17, 44, 45]. This will allow the use of computers and computing resources in order to gain new biological insights. This is a great challenge, especially given noise and hidden variables, and is a subject of our future work. We hope that the development of theoretical models and tools, such as the one presented here, will facilitate research in this important direction.

## Methods

### Stochastic simulation

eSTG programs can be naturally simulated by the well-known Gillespie stochastic simulation algorithm [6]. Gillespie's implementation uses the rates of all possible reactions and chooses stochastically the next reaction by assuming that the time to the next reaction is exponentially distributed with rate parameters corresponding to the reaction rates.

A rule of the form:


can be converted into *n* separated reaction rules:


and thus existing implementations of the Gillespie algorithm can be used to determine the next reaction and the time interval. Applying these rules to build the lineage tree is described in the Operational semantics section.

The code that was used to generate the examples in this paper will be made available as an open source tool and is currently under preparation for publication.

### Equivalence and conversion to other languages

In this section we compare the expressiveness of eSTG to 4 other families:

1. maODE: Ordinary Differential Equations arising from mass-action kinetics.
2. maCRN: Chemical Reaction Networks with mass-action kinetics.
3. gCRN: Chemical Reaction Networks with general rate kinetics.
4. U-gCRN: Unimolecular Chemical Reaction Networks with general rate kinetics.

An maCRN is a chemical reaction network where each reaction has an associated rate constant, and where the instantaneous rate of a reaction is determined by the product of the rate constant with the instantaneous concentrations of the reagents. It is known that an maCRN under that mass-action law produces a system of ODEs with a special structure, here called an maODE system. In an maODE system each right-hand-side of each differential equation for species *s* has the form of a polynomial over the set of species, where each monomial with a negative sign has *s* as a factor (raised to some non-zero power). Conversely each maODE determines a canonical maCRN that has that maODE as its kinetics. Therefore there are canonical translations back and forth between maODEs and maCRNs [46].

A gCRN is instead a Chemical Reaction Network where each reaction has an associated rate function from current or past system states to changes of concentrations. The instantaneous rate of a reaction is then given immediately by its rate function without further considerations; the class of ODEs that a gCRN may generate depends on the class of rate functions that are available.

A U-gCRN is a special case of a gCRN where all the reactions are unimolecular. For sufficiently powerful rate functions it is possible to have a (nominally) unimolecular reaction depend on the concentrations of other species, so that U-gCRN is in fact as expressive as gCRN. For example, an maCRN reaction  can be translated to the gCRN reaction  where [*A*] is the instantaneous concentration of *A*, and an maCRN reaction  can be translated to a gCRN reaction  or to two U-gCRN reactions  and .

The family of population dynamics specifications that can be described using basic eSTG is equivalent to U-gCRN. A U-gCRN reaction  can be translated into an eSTG reaction , and conversely an eSTG reaction  can be translated into a set of U-gCRN reactions .

The U-gCRN form of eSTGs implies that one must make choices in modelling: the main species that are the focus of a model, and occur in the left-hand side of productions, will be reflected in the generated lineage trees, but auxiliary species that appear only in the rate laws will not, even when those would be considered as equal in a model based on bimolecular interactions.

### Operational semantics

We will start with basic definitions for the semantics of Lineage Trees. Paths in a tree are represented as finite sequences of natural numbers *π* = *n*_1_, …, *n*_*m*_ ∈ ℕ* (star means finite sequence, with *nil* as the empty sequence, and ", " as sequence concatenation). Each number *n* in a path represents the *n*^*th*^ child of a node, starting from the root. Nodes in a tree are labeled by an alphabet *S*_0_ = *S* ∪ {0} consisting of species in *S* and a distinguished symbol 0 ∉ *S* (the "dead" leaf).

*Definition*: a *tree L* is a partial function in ℕ* → *S*_0_, from paths in ℕ* to label nodes in *S*_0_, whose domain is non-empty and prefix-closed (that is, L(*π*_1_, *π*_2_) defined ⇒ L(*π*_1_) defined).

*Definition:* A *leaf* in a tree *L* is a maximal path *π* - one such that *L*(*π*) is defined and there is no *π* ' ≠ *nil* where *L*(*π* , *π*') is defined. We also say that *π*, *B* is a (*B* -labeled) leaf in *L* if *π* is a leaf in *L* and *L*(*π*) = *B*.

*Definition*: A *lineage tree L* is a tree where each path *π* such that *L*(*π*) = 0 is a leaf.  is the set of such trees.

By these definitions, a tree is a non-empty set of paths and each node has a "unique label" which is the path *π* that leads to it. A root-only tree is a function from *nil* to some species *A*.

Next, we use the *λ* -calculus notation for the definitions of lineage tree operators (if *f* (*x*) = *b* then we write *f* = *λx. b*). We use the element *undef* for partially defined functions:

1. The lineage tree with just one dead leaf:
2. The lineage tree with root *A* ∈ *S* and children *L*_*i*_, for *A* ≠ 0 and *n* > = 0:

where for *n* = 0, *A* = *A*() is a "live" leaf.

3. The *leaf-extension* operator *L*, *π*, *A* ⊲ (*B*_1_, … *B*_*n*_), which is defined if *π* is an *A* -labeled live leaf in *L*(*L*(*π*) = *A* ≠ 0), and *n* > 0, and *B*_1_ … *B*_*n*_ ∈ *S*_0_:

For example, by the above definitions a tree with root *C* and with *n* children *B*_1_, …, *B*_*n*_ which are all leaves can be written as the expression *C*(*B*_1_(), …, *B*_*n*_()), representing a function that given the sequence *nil* returns the label *C*, given the sequence *i*, *nil* returns the label *B*_*i*_, and is otherwise undefined. Similarly, the expression *C*(), *nil*, *C* ⊲ (*B*_1_, …, *B*_*n*_) represents the tree *C*() where the leaf *C* is extended into a node with children *B*_1_, …, *B*_*n*_; this is then the same as the tree *C*(*B*_1_(), …, *B*_*n*_()).

A collection of eSTG reactions describes a way of generating and transforming lineage trees. We now describe how each eSTG reaction transforms a lineage tree into new lineage trees. More precisely, since eSTG reactions are stochastic/probabilistic, how each reaction produces a *measure* of new lineage trees, where each new lineage tree is associated with its rate of occurrence.

*Definition*: A *measure*is a function from finite tuples of lineage trees to non-negative reals, with operators:


the singleton measure, which measures  as *r* and everything else as 0;


the sum measure, with *m* > 0;


the leaf-extension measure, where *π*, *B* is a leaf in *L*; this is a function in . This is the measure such that any extended tree of the form *L*, *π*, *B* ⊲ (*L*_1,_ …, *L*_*n*_) for some *L*_1_, …, *L*_*n*_ receives the measure *M*(*L*_1_, …, *L*_*n*_).

For example, *C*(), *nil*, *C* ⊲ (*d*(*r*, (*D*(), *E*())) + *d*(*s*, *F*())) = *d*(*r*, *C*(*D*(), *E*())) + *d*(*s*, *C*(*F*())) because *C*(*D*(), *E*()) has the shape *C*(), *nil*, *C* ⊲ (*D*(), *E*()) and so it receives measure *r*, and *C*(*F*()) has shape *C*(), *nil*, *C* ⊲ (*F*()) and so it receives measure *s*.

We are now ready to define the effect of a set of eSTG reactions ***S*** on lineage trees. This is given as a *reduction* relation ***R*** between lineage trees and measures. We write *L* → *M* (*L reduces to M*) for (*L*, *M*) ∈ ***R***, where ***R*** is defined as the smallest relation satisfying the following rule:


This rule prescribes, for example, how to carry out a simulation of a set of eSTG reactions given an initial lineage tree: at each step apply the rule above to all applicable reactions and tree leaves, sum all the measures so obtained, and sample a new lineage tree according to the resulting measure.
